# Implants Placement in Contact with Dental Tissue: A Potential Paradigm Shift? Systematic Literature Review

**DOI:** 10.1055/s-0039-1697213

**Published:** 2019-12-31

**Authors:** Amel Labidi, Sana Bekri, Lamia Mansour, Sonia Ghoul-Mazgar

**Affiliations:** 1 Department of Removable Prosthodontics, Faculty of Dental Medicine, University of Monastir, Monastir, Tunisia; 2 ABCD F Laboratory of Biological, Clinical and Dento-Facial Approach, University of Monastir, Monastir, Tunisia; 3 Laboratory of Histology and Embryology, Department of Oral Histology, Faculty of Dental Medicine, University of Monastir, Monastir, Tunisia

**Keywords:** dental implants, tooth, impacted, tooth root, tooth ankylosis, prosthodontics

## Abstract

The aim of this study was to explore the literature for clinical and histological data of an unconventional treatment with implants placement in contact with dental tissue (IPICDT) and to try to clarify its indications and surgical procedure particularities.Relevant publications published until May 2019 on the IPICDT were thoroughly reviewed. Search strategy was developed using a controlled vocabulary combination.Medline’s exploration and manual research identified 397 articles; 15 of these were selected after screening. IPICDT was indicated in three clinical situations: impacted teeth, ankylosed teeth, or residual roots. Clinical and radiological follow-up were satisfied except for implants placed in contact with (and not through) roots. Histological analysis revealed different mineralized tissues formed on the implant surface: cementum on removed implants in human and osteodentin on implants placed in contact with animal teeth dentin and pulp. These findings were described as new concept of implants’ “Mineral integration.”According to this study, the follow-up results of implants placed in contact with roots were controversial. Some implants were stable and others were either removed or kept and disinfected after root extraction because of bacterial infection. However, implants placed through ankylosed or impacted teeth were stable. These findings suggest that the clinicians have to be cautious when applying this unconventional approach. Further studies are recommended to explore its long follow-up. It is also interesting to explore this technique in cases of syndromic dental diseases with several impacted teeth (such as cleidocranial dysplasia; or amelogenesis imperfecta).

## Introduction

In implantology, several changes have been introduced since the basic concepts proposed by Branemark. 1 Among these changes, implant connection, one-time surgery, and immediate loading could be cited. 2 3 The only common concept that has not been changed is the concept of osteointergration, where the implant surface is intimately covered with bone. However, in some clinical situations particularly when the teeth are impacted, it seems critical to indicate implants. In fact, the surgical removal of the teeth seems to compromise the bone tissue. Considering the anterior region, the aesthetic rehabilitation of previously damaged sites often requires additional surgical procedures that are complex, time consuming, and expensive. 4 Several papers published in the literature explored the possibilities of the contact of implants with other tissues than bone. However, these studies were essentially in vivo performed on animals and the results were different. Also, there was no consensus concerning this subject.

The aim of this study was to explore the literature to analyze the clinical data of an unconventional treatment with implants placement in contact with dental tissue and to try to assess its indications and surgical procedure particularities.

## Methods

The literature search for relevant articles was performed in MEDLINE database using PubMed.

The search strategy was developed using the terms “Dental Implants” [Mesh]; “Tooth, Impacted” [Mesh]; “Tooth Root” [Mesh], and “Tooth Ankylosis” [Mesh]. These Mesh Terms were combined in the following Boolean equations: “Dental Implants” [Mesh] AND “Tooth Impacted” [Mesh]; “Dental Implants” [Mesh] AND “Tooth Root” [Mesh]; “Dental Implants” [Mesh] AND “Tooth Ankylosis” [Mesh]. The final update of the search was checked on April 2019. A manual research was also performed.

Only the findings described by the authors in these papers were considered. Clinical photographs or radiographs were not used to include additional findings. Data were collected by two independent reviewers using a pre-established checklist for data collection. In case of disagreements, consensus was achieved by discussion among the reviewers.

## Results

### Articles Selection

A total of 397 articles were found; 391 of them using PubMed and six manually.

**Fig. 1:**
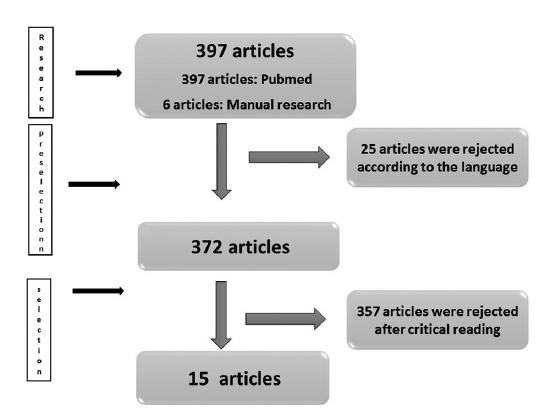
Diagram of selection of the articles.

Considering the exclusion criteria, 25 articles were excluded because of the language (other than English and French), and 357 articles after reading for several reasons. Fifteen articles were finally withheld according to the diagram of article selection ( Fig. 1 ). Concerning the type of study; one article presented a cohort study; eight were case reports or case series, and six articles concerned animal experimental studies.

### Clinical and Radiological Data

The implant placement in contact with dental tissue was indicated in three clinical situations: impacted teeth 5 6 7 8 ; ankylosed teeth 9 10 ; and residual roots. 6

#### Ankylosed Teeth

This unconventional implantation protocol was practiced at first on ankylosed anterior teeth. 9 For five patients with ankylosed teeth, five implants were placed intentionally through root fragments that were left in the osteotomy site. However, for one patient, an implant was placed unintentionally in contact with an ankylosed retained molar root fragment.

For the five implants placed through anterior ankylosed roots, whatever the mode of healing, i.e., submerged, nonsubmerged, or immediately loaded, they healed without incident. Clinical stability was achieved at the end of the integration period of 3 to 7 months. The conventional prosthetic steps were undertaken and the prostheses were delivered within a month. Clinical and radiological follow-up ranged from 1 to 3.5 years. Signs of limited resorption of dentin have been observed on one implant: The most coronal portion of the remaining dentin appeared to be involved in a remodeling process, similar to that occurring on the opposite mesial side with a bone interface. This was attributed to local implant overload. For the rest of the implants placed in contact with ankylosed teeth, they showed no particular modification, neither on the bone-implant interface nor on the implant-root interface.

Concerning the implant placed unintentionally in contact to the ankylosed molar root fragment, it remained in function for 4 years without any problems. Then it was removed for clinical mobility. Radiographic control revealed peri-implant radiolucency ( Table 1 ).

**Table 1:** Implants placed through ankylosed teeth

Number of patients	Sex	Age (year)	Dental site	Healing unloaded period (month)	Total follow-up (month)	Bone-implant interface	Root-implant interface	Modification of dentine fragment
1	M	52	11	7	49	Normal	Normal	Partial resorption
1	F	40	11	7	45	Normal	Normal	No visible change
1	M	59	42	3	27	Normal	Normal	No visible change
1	M	34	11	6	27	Normal	Normal	No visible change
1	F	40	11	–	12	Normal	Normal	No visible change
1	F	56	46	–	48	Peri-implant radiolucency	Peri-implant radiolucency	–

#### Impacted Teeth

Transdental implants were also used in the cases of impacted teeth. 5 6 7 8 A total of 20 patients were treated with 32 implants placed through impacted canines and premolars. The follow-up was for a period ranging from 6 months to 8 years.

No postoperative pain was noticed even when the implant is placed through the pulp chamber. The implant healing period was silent except for one implant which presented soft tissue inflammation 15 days later. It was successfully treated with antibiotics. One case of failure has been reported: a short implant 8.5 mm, placed in contact with the root of an impacted canine, was lost after 4-months. 5

Clinically, all implants were stable. Radiologically, all implants presented successful healing, except for one. It presented bone loss on the mesial side due to the small distance separating it from the adjacent tooth. However, clinically the implant was stable ( Table 2 ).

**Table 2:** Implants placed intentionally through impacted teeth

Number of patients	Age (years)	Sex	Implant number	Dental site	Implant type	Dental tissues in contact with the implant	Complications	Success rate	Follow-up duration
1	62	F	3	13, 23	Nobel biocare Osséotitebiomet 3i 3.75*11.5 3.75*8.5 XP 4 /5*15	2 implants (C ^‡^ ) (D ^†^ ), (P ^§^ ) 1 implant (C), (D)	–	±	A short implant (8.5 mm) was lost after 4 months All the other implants were stable after 4 years
1	31	F	1	23	NT osseotite e Ø 5 15 mm﻿	P; Radicular D	–	+	6 months:implant stable Satisfiying radiological control: 4 and 8 years
1	80	M	3	PM-M	1 osseotite Ø 4/5 13 mm﻿ 2 full osseotits Ø 5 /11.5 Ø 4 /13	2 (E*, D, P) 1 (D, C, P, periodontal ligament)	–	+	Successful follow-up after 2, 6, and 8 years
5	ns	Ns	7	ns	Ns	ns ^||^	–	+	Successful follow-up after 6 months and 3 years
1	32	F	2	13, 23	Nobel replace Ø 5 /13MM	E, radicular D, P	–	+	Successful follow-up after 6 months and 3 years
1	33	F	1	13	OsseotiteNT Ø4.3*13 mm﻿	E, D, P	–	+	Successful follow-up after 8 years
1	80	M	3	31, 33, 34	3 OsseotiteNT *a* Ø4.3*13 mm﻿	C, D, P -E, D, P -E, D	–	+	Successful follow-up after 5 years
1	85	F	1	13	Nanotiteosseotitea Ø4.0* 10 mm﻿	C, D, P	–	+	Successful follow-up after 5 years
1	71	M	1	13	OsseotiteNTa Ø4.3*13 mm﻿	C, D, P	–	+	Successful follow-up after 3 years
1	64	F	1	23	Nobel Activeb Ø4.3*13 mm﻿	C, D, P	–	+	Successful follow-up after 3 years
1	58	M	1	13	Nobel Activeb Ø4.3*13 mm﻿	E, D, P	–	+	Successful follow-up after 2 years
1	32	F	2	13, 23	Nobel Activeb Ø4.3*13 mm Replace Ø4.3*13 mm﻿	1 (C, D, P) 1 (E, D, P)	–	+	Successful follow-up after 1.5 years
1	66	F	2	13, 12	2 Nobel Activeb Ø3.5*13 mm﻿	E, D, P	–	+	Successful follow-up after 5 years
1	55	F	1	23	Replaceb Ø3.5*15 mm﻿	E, D, P	–	+	Successful follow-up after 1 years
1	69	M	2	23, 24	2 Nobel Activeb Ø4.3*13 mm﻿	C, D, P E, D, P	–	+	Successful follow-up after 1 years
1	85	F	1	13	ns	–	–	+	Successful follow-up after 5 years
Note: *E: Enamel; ^†^ D: Dentin; ^‡^ C: Cementum; ^§^ P: Pulp; ^||^ ns: not specified; M, molar; PM, premolar.									

#### Residual Roots

Implant placement through residual roots was also described. Six patients were treated with seven implants placed intentionally across a root. 11 For nine patients, implants were placed unintentionally in contact with residual root fragments. 10 12 13

For the implants placed through the roots, residual root fragments were clinically and radiologically asymptomatic and covered with bone or gingiva. The presence of an endodontic material did not affect the decision to encroach on the root fragment and the reasons for tooth extraction were not justified.

The healing period (3–6 months) after surgical procedure was respected before the conventional prosthetic steps were undertaken.

All implants were clinically and radiographically monitored from 3 to 9 years

No implant failed during this period.

In one case; radiographic follow-up after 9 years showed bone loss at the second and third implant threads. The vertical bone defect observed was similar in both sides of the implant: the side in contact with the root and the one in contact with the bone.

Otherwise, usual radiographic features were observed at the root-implant interface ( Table 3 ).

**Table 3:** Implants placed intentionally through residual roots 11

Patient number	Sex	Age (years)	Dental site	Characterization of the residual root	Description of the local situation	Endodontic material	Last radiographic follow-up	Bone-implant interface	Root-implant interface	Modification of root fragment
1	F	66	36	1/2 apical root	Residual root covered by bone, PDL visible	Partial endodontic material	9 years	Normal	Normal	No visible change
1	F	66	37	1/2 apical root	Residual root covered by bone, PDL visible	Partial endodontic material	9 years	Normal	Normal	No visible change
2	F	59	45	2/3 apical root	Residual root covered by bone, PDL visible	Endodontic material	20 months (6 years)	Normal	Normal	No visible change
3	M	62	11	1/3 apical root	Residual root covered by gingiva, PDL visible	No endodontic material	5 years	Normal	Normal	Possible partial resorption
4	F	44	15	2/3 palatal root	Residual root covered by bone, PDL visible	Endodontic material	3 years	Normal	Normal	No visible change
5	M	51	24	2/3 apical root	Residual root covered by bone, no visible PDL	No endodontic material	3 years	Normal	Normal	No visible change
6	F	18	45	Tip of mesial root	Remained root of ankylosed deciduous molar, no visible PDL	No endodontic material	3 years	Normal	Normal	No visible change
Abbreviation: PDL, periodontal ligament. Source: Adapted with permission from Szmukler-Moncler et al. 11										

Concerning the implants placed unintentionally in contact with residual root fragments; nine implants were placed. Several implant systems were used. Follow-up ranged from 6 months to 10 years. Five implants were removed. Patients consulted essentially for the implant mobility. For the other implants, they were kept in place and disinfected after residual root extraction ( Table 4 ).

**Table 4:** Unintentional implants placed in contact with residual roots 13

Patient	Sex	Age (years	Dental site	Reason for tooth extraction	Endodontic material	Time from initial implant fragment placement to root discovery (month)	Implant removed
1	F	59	36	Fracture	Yes	21	Yes
2	M	74	37	Fracture	Yes	25	No
3	M	74	35	Periodontal disease	Yes	12	No
4	F	59	25	Fracture	Yes	48	No
5	M	68	12	Caries	Yes	13	Yes
6	F	70	46	Caries	Yes	6	No
Source: Reprinted with permission from Langer et al. 13							

#### Surgical Protocol Particularities

When the implant was intentionally placed in contact with dental tissue, a computed tomography (CT) examination was performed. The diameter of the implant and its length were selected to respect the classical principles of implant placement. The implant was placed in its prosthetically required position.

If the implant was placed through the coronal part of the tooth, the drilling using a turbine-mounted tungsten carbide bur was performed to open a channel through the coronal enamel. Then, the expansion of the implant placement was done with the standard drilling tool; the pilot drill brings back dentin.

If the implant is placed through the root, the implant standard drills were used.

Thread tap were used to facilitate implant placement because the walls of the cavity in contact with the dental tissue were rigid. 8

### Histological Data

The histological findings concerning the tissue formed around implants in contact with dental tissue were explored essentially through animal studies. 14 15 Three case reports/case series explored histologically the neoformed tissue in human. 12 16

#### Histological Data from Animal Reports

The animal studies explored the tissue formed in case of implants placed just in contact with roots. Authors revealed the formation of mineralized tissue described as a cementum on the implant surface. In addition, the formation of a gap between this neocement and the adjacent bone surface with blood vessels and fibers was described.

Polarized light microscopy revealed that connective tissue fibers had varied orientations, either perpendicular or parallel to the implant surface, and mostly with an insertion at the neocement formed on the surface of the implant. 17 This fact was in contradiction with human histological findings. 12

Authors also explored the interface between implant and dental tissues when the implant is placed through the root.

A hard tissue layer established a close contact with large areas of the adjacent titanium surface.

Schwarz et al histologically explored this formed hard layer. In fact, on the exposed coronal pulp, thick layers of reparative dentin were formed. The dentine was tubular when observed in contact with the pulp, atubular when explored at the implant surface. In addition, a compact layer of osteodentin filled the gap between the implant surface and the exposed coronal dentin.

The osteodentin formed in the contact zone was superimposed by a thin layer of osteocementum. This osteocementum appeared to have a higher mineral content. 16 These data were in accordance with Gray JL et al findings. A layer of hard tissue, identified as neocement, had formed on large areas of the implant surface in contact with roots or dental tissue. In some areas, a cementation bridge from the root to the implant was described. 18

#### Histological Data from Human Reports

Guarnieri et al explored an implant placed in contact with a residual root that was lost for peri-implantitis after 8 years of function. The histological analysis revealed cementum formation on the implant surface. However, the space between the root and the implant was present in the form of a rudimentary space with a continuous layer of cementum strongly adhering to the surface of the implant, with no blood vessels or collagen fibers. 12 These findings were in line with another case of an implant placed in contact with a root fragment, that was removed after 11 years for mobility. Histological analysis revealed cementum in contact with the retained root surface with no sign of periodontal ligament. There was also graft material and newly formed bone between the implant and the root surfaces in some places. In addition, the presence of a thick biofilm, calculus formation, and extensive bacterial infiltration on the implant surface were observed. 13

Two cases of implants were placed in contact with buccal root fragments. The roots were at first not radiologically detected. The implants were kept in place, disinfected and the residual roots were removed and histologically analyzed. The external root fragment surfaces presented cementum and periodontal ligament (PDL) peripherally. Authors did not precise if the root surface with PDL was in contact with the implant surface or not. The middle part of the root fragments presented viable bone tissue without any significant inflammatory component. 10

## Discussion

The results of the literature search showed three indications for IPICDT use: ankylosed teeth, then the residual dental roots, and the impacted teeth.

In the first case of ankylosed teeth, the idea was to preserve the maximum of bone for implants because the extraction of ankylosed tooth could have been complex. 4 Clinically, asymptomatic sites free from inflammation before treatment were chosen. The fate of the remaining root fragment was a source of concern. It may remain asymptomatic or it may be resorbed and substituted with bone. 9 So, longer follow-up is necessary to explore the evolution of the tissue in contact with implants.

In the case of implants placed through the residual roots, the follow-up outcome is controversial. In fact, the implants placed intentionally through residual roots were stable. However, late dental implant failures were described when they were placed unintentionally in contact or in close proximity with residual root fragments. This could be due to the fact that teeth are usually extracted for periododontal diseases or endodontic failure commonly accompanied with bacterial contamination. This could affect the implant healing and integration. 13

Histological data showed neocement formation on the implant surface in contact with root. The newly formed cementum is suggested to be derived from the progenitor cells of the dento-periodontal ligament and not the cells of the alveolar bone. 17 19

The cement adheres perfectly to the implant surface in human with colonization of the rough implant surface by cementocytes. This hypercementosis is possibly a biological reaction due to the inflammatory stimulus.

In the case of impacted teeth, a short implant placed in contact with an impacted canine was lost. According to the author, longer implants would have been placed, without hesitation to cross the pulp chamber. The lost implant would probably have kept its clinical stability. The implants placed through dentin and pulp chamber were stable. This could be explained by the formation of tertiary dentin originated from potential differentiation of pulp stem cells. Bone sialoprotein is a major component of tertiary dentin. It has a close similarity to bone. This could explain the close adhesion of tertiary dentin to the implant. In addition, the impacted teeth are free of periodontal and endodontic inflammation. Further, histological human studies are necessary to confirm these hypotheses, 13 and to explore the healing process in case of structural anomalies. 20

No postoperative pain was noticed even when the implant crossed the pulp. This could be explained by the absence of bacterial infection in these teeth. 21

## Conclusion

More cases need to be documented demonstrating long-term and postloading results in humans before intentional tooth/root contact might be considered a reliable clinical option suitable for general use. The unconventional protocol of placing implants in contact with dental tissue was tried by several authors. Implants placed through impacted and ankylosed teeth were stable. However, clinical and histological findings of implants placed in contact with residual roots were controversial, but encouraging. This unconventional protocol opens a new treatment option for edentulous sites because of the presence of a single impacted tooth like a canine for example, but also applies to other indications with several impacted teeth, such as for syndromic dental diseases like cleidocranial dysplasia or amelogenesis imperfect; to avoid invasive surgeries by placing implants through dental tissue. However, before considering intentional tooth/implant contact in general use, more cases are needed to be documented and further studies are recommended to demonstrate long-term, postloading results in humans.

### Note

The manuscript has not been published or submitted elsewhere. The manuscript has been read and approved by all the authors.
